# Optocapacitance: physical basis and its application

**DOI:** 10.1007/s12551-022-00943-9

**Published:** 2022-04-13

**Authors:** Bernardo I. Pinto, Carlos A. Z. Bassetto, Francisco Bezanilla

**Affiliations:** 1 Department of Biochemistry and Molecular Biology, The University of Chicago, Chicago, IL 60637 USA; 2 Centro Interdisciplinario de Neurociencia de Valparaíso, Facultad de Ciencias, Universidad de Valparaíso, Valparaíso, Chile

**Keywords:** Optocapacitance, Membrane capacitance, Remote neuronal stimulation, Nanoparticles, Temperature

## Abstract

The observation that membrane capacitance increases with temperature has led to the development of new methods of neuronal stimulation using light. The optocapacitive effect refers to a light-induced change in capacitance produced by the heating of the membrane through a photothermal effect. This change in capacitance manifests as a current, named optocapacitive current that depolarizes cells and therefore can be used to stimulate excitable tissues. Here, we discuss how optocapacitance arises from basic membrane properties, the characteristics of the optocapacitive current, its use for neuronal stimulation, and the challenges for its application in vivo.

## The nature of membrane capacitance and the equivalent circuit of the membrane

**Fig. 1:**
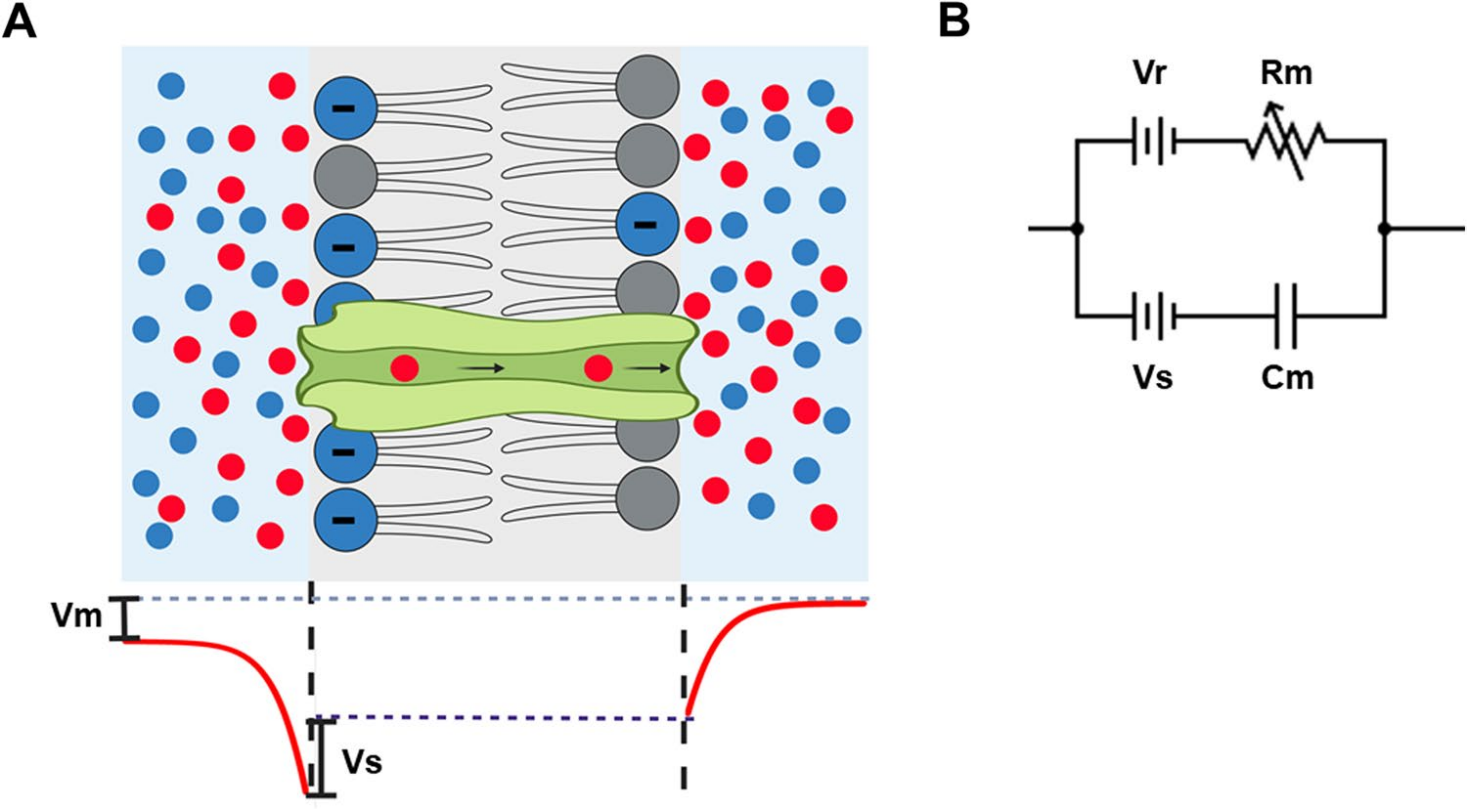
**A** Diagram of the major components of the membrane. Ion channels provide a pathway for the selective transport of ions. The bottom shows the electrical potential perpendicular to the membrane where negative charges of the phospholipid components generate a surface potential difference (*Vs*). **B** Electrical equivalent circuit including the membrane capacitor (*C*m), surface potential (*Vs*), membrane resistance (*Rm*), and battery associated with ionic conductance (*Vr*). Variable resistance is used for *Rm* due to the opening and closing of ion channels

The change in membrane capacitance generated by heating due to absorption of light, also known as the optocapacitive effect, arises from the physical properties of the membrane. The plasma membrane separates the interior of the cell from the outside environment and is composed mainly of a phospholipid bilayer in which proteins are embedded. The hydrophobic core of the membrane prevents the diffusion of charged molecules such as ions from one side of the membrane to the other. This electrical insulation between two conducting media is alike a parallel plate capacitor. The presence of charged groups in the phospholipids generates a surface charge, which produces a surface potential on each face of the membrane. That, in turn, will attract ions from the solution and change the effective local concentration of ions near the membrane that will screen the membrane surface charge (Fig. 1a) (McLaughlin 1989). The surface potential is mainly affected by the concentration and valence of the ions in the solution. Polyvalent ions are more effective than monovalent ions at screening the surface charge. The asymmetric composition of the plasma membrane with more negatively charged phospholipid in the cytoplasmic face produces an asymmetric surface charge distribution, and thus a surface potential difference (*Vs*) between the internal and external leaflet (Daleke and Huestis 1985; Seigneuret and Devaux 1984).

The membrane bilayer can be conceptualized electrically as a capacitor (*C*m) that is in series with a battery whose voltage is given by *Vs*. Ion channels and transporters present in the plasma membrane provide a pathway for ionic conduction between the internal and external environment. In the membrane equivalent circuit, they can be modeled as resistors (*Rm*) in series with their own batteries (*Vr*), which is given by the reversal potential of the conducting ions. These components are in parallel with *C*m and *Vs* (Fig. 1b).

## Physical basis for thermal dependence of membrane capacitance

In this section, we will use a very simple model to show how the temperature dependence of the cell capacitance can be easily deduced. The capacitance (*C*) of a parallel plate capacitor is given by the following relation:

1 $$C=\frac{\varepsilon A}d$$

where *A*, *d*, and *ε* are, respectively, the area of the plates, the separation between the plates, and the relative permittivity of the material between the plates, in this case, the lipidic bilayer. From this simple equation, we can see that capacitance can be affected in three ways: by changing the permittivity (1), the area (2), or the thickness (3) of the capacitor.

Considering the thermal expansion of the bilayer, increasing the temperature of the membrane bilayer will increase its area by the following relationship:

2 $${A}_{2}=l\left(1+\alpha ({T}_{2}-{T}_{1})\right)*w \left(1+\alpha {(T}_{2}-{T}_{1})\right)$$

where *A*_2_ is the area at temperature *T*_2_, *α* is the expansion coefficient of the bilayer, $l$ and $w$ are the length and the width that define the area at *T*_1_. Similarly, the thickness of the membrane will also be affected by temperature as follows:

3 $${d}_{2}={d}_{1}\left(1+\alpha{(T}_{2}-{T}_{1})\right)$$

where *d*_1_ and *d*_2_ are the thickness at temperature *T*_1_ and *T*_2_, respectively.

Rewriting Eq. 1 to accommodate the final capacitance (*C*_2_) at *T*_2_ and using Eqs. 2 and 3, we get

4 $$C_2=\varepsilon\left(\frac{l\left(1+\alpha\triangle T\right)w\left(1+\alpha\triangle T\right)}{d_1\left(1+\alpha\triangle T\right)}\right)$$

5 $$C_2=\varepsilon\left(\frac{lw\left(1+\alpha\triangle T\right)^2}{d_1\left(1+\alpha\triangle T\right)}\right)=\varepsilon\left(\frac{lw}{d_1}\right)\left(1+\alpha\triangle T\right)$$

where Δ*T* is the difference between *T*_2_ and *T*_1_.

We can finally obtain the relationship between the initial capacitance (*C*_1_) at *T*_1_ and the capacitance at any *T*_2_:

6 $$C_2=C_1\left(1+\alpha\triangle T\right)$$

This very simple model correctly predicts the linear dependence of the membrane capacitance with temperature, which has been theoretically predicted (Plaksin et al. 2018) and experimentally observed in a wide variety of preparations (Carvalho-de-Souza et al. 2015; Martino et al. 2015; Pinto et al. 2021; Shapiro et al. 2012; Taylor 1965). Nevertheless, this is a model and should be considered as such. The reality poses a more complex picture.

**Fig. 2:**
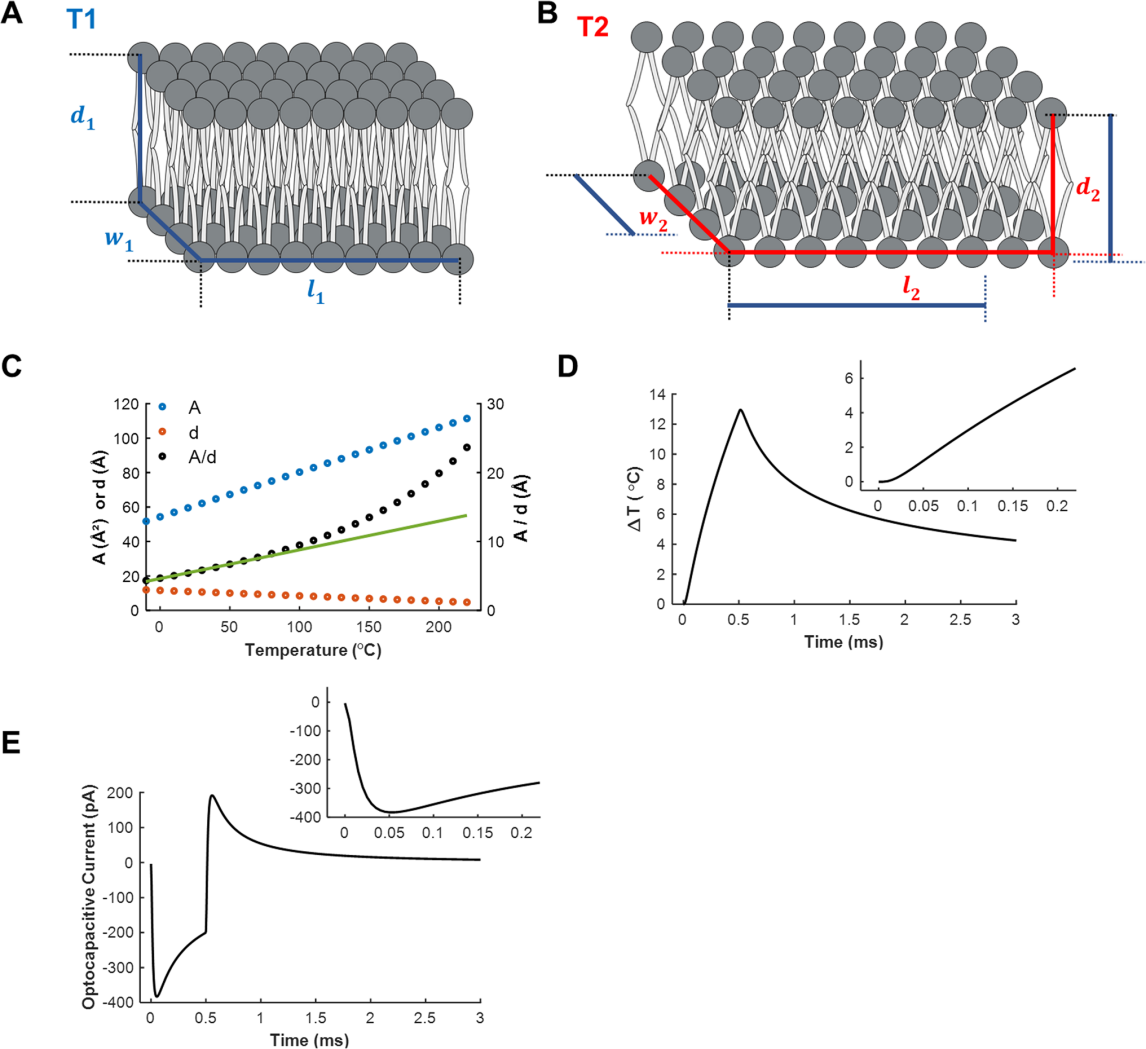
Thermal effect on the membrane and its role in the generation of optocapacitive currents. **A** and **B** represent the membrane geometry at temperatures *T*_1_ and *T*_2_, respectively (T_2_ > T_1_). *W*, *d*, and *l* are the width, length, and thickness of the membrane, respectively. The bars indicate the dimension of *w*, *d*, and *l* at *T*_1_ (blue) and *T*_2_ (red). Note the increase in *w* and *l* and the decrease in *d* with temperature. **C** Average area (*A*, blue), thickness (*d*, red), and the ratio between the area and thickness of a lipid in a bilayer (*A*/*d*, black). *A*, *d*, and *A*/*d* show a linear relationship with temperature in the relevant biological range (green line for *A*/*d* in the 0 to 60 °C range). The values of thickness (*d*) and area (*A*) as a function of temperature were fitted with linear equations ($A=A_1\left(1+\alpha_A\triangle T\right)$ and $d=d_1\left(1+\alpha_d\triangle T\right)$, respectively) using experimental values from DLPC bilayer by Petrache et al. 2000 (data taken from Table 5). The fitted equations parameters were $\alpha_A=4.8\ast10^{-3}\;^\circ\mathrm C^{-1}$, A_1_ = 54 Å^2^, $\alpha_d=-2\ast10^{-3}\;^\circ\mathrm C^{-1}$, and d_1_ = 12 Å considering ${A}_{1}$ and ${d}_{1}$ at *T* = 0 °C. Values for *A*, *d* and *A*/*d* were calculated from the fittings. **D** and **E** are the simulated temperature change and elicited capacitive currents respectively. Inset shows an expanded time window

Determinations of bilayer structure can provide insights into the physical mechanism underlying the temperature dependence of membrane capacitance. Using nuclear magnetic resonance techniques, it has been shown that the thickness of the membrane decreases with temperature, and the area per lipid increases (Petrache et al. 2000). Similar results have been found in charged membranes using solution small-angle X-ray scattering (Szekely et al. 2011). These results are at odds with the solid parallel plate model for the membrane capacitor (Fig. 1B, Eqs. 2 and 3) since from thermal expansion theory both the area and the thickness should increase. This exquisite feature of the membrane has been attributed to the trans-gauche rotational isomerization of the phospholipids hydrocarbon chains, which has a large thermal dependence and results in an increase of the angle between the lipid’s tails (Fig. 2A and B) (Kučerka et al. 2011). We can incorporate this anisotropic expansion of the bilayer into the previously built model. From experimental measurements (Petrache et al. 2000), the area and thickness of the bilayer have a linear relationship with temperature; therefore we can rearrange Eq. 4 to get:

7 $$C_2=\varepsilon\left(\frac{A_1\left(1+\alpha_A\triangle T\right)}{d_1\left(1+\alpha_d\triangle T\right)}\right)$$

where ${\alpha }_{A}$ and ${\alpha }_{d}$ are the expansion coefficient for the area and thickness of the bilayer, respectively. Due to the decrease of thickness with temperature ${\alpha }_{d}$ is negative whereas ${\alpha }_{A}$ is positive. Even though Eq. 7 is nonlinear, using the experimentally measured expansion coefficients for DLPC (Petrache et al. 2000), we find that over the range of temperature between 0 and 60 °C, the ratio between area and thickness *(A/d)* has a linear relationship (Fig. 2C). This will produce a linear increase of *C* with *T* given that $\varepsilon$ does not change with *T*. Nevertheless, it has also recently been proposed that a major contribution for the temperature dependence of the capacitance is given by water penetration in the plasma membrane, which would increase its permittivity (Bondelli et al. 2021). This increase in permittivity would make the relation between capacitance and temperature even more nonlinear. Therefore, we are still missing a theoretical description, based on the molecular structure of the lipid bilayer, capable of reproducing the observed linear relationship between membrane capacitance and temperature in the biologically relevant temperature range.

## Optocapacitive currents explained

We have shown above, in a simplistic way but also based on experimental measurements, how a change in temperature will lead to a change in capacitance of the plasma membrane. In order to understand the relationship between changes in capacitance and the resulting currents, let us consider the charge stored by the capacitor in the circuit of Fig. 1B. Upon steady-state conditions, the charge stored by this capacitor is:

8 $$Q=C*\left(Vm-Vs\right)$$

where *Vm* is the membrane voltage, not to be confused with *Vs*.

We can obtain the current flowing in the membrane as the derivative of the charge with respect to time.

9 $${I}_{C}=\frac{dQ}{dt}=\left(Vm-Vs\right)\frac{dC}{dt}+C\frac{{dV}_{m}}{dt}$$

Thus, under voltage-clamp conditions $(\frac{{dV}_{m}}{dt}=0)$, we can infer that a change of capacitance in time will produce a current. The amplitude of this current will be linear with voltage, and proportional to the difference between *Vm* and *Vs* as well as the rate of change of the capacitance, as follows:

10 $${I}_{C}=\left(Vm-Vs\right)\frac{dC}{dt}$$

Due to the linear relation of capacitance with temperature we can substitute the Eq. 6 in 10 to obtain

11 $$I_C=\left(Vm-Vs\right)\frac{d(C_1\left(1+\alpha\triangle T\right))}{dt}$$

Therefore, the optocapacitive current is given by

12 $${I}_{C}=\left(Vm-Vs\right){ C}_{1} \alpha \frac{dT}{dt}$$

It is important to note that the optocapacitive current follows the derivative of the temperature over time. To illustrate the optocapacitive currents, we modeled the temperature change and the resulting currents induced by a homogeneous heat source placed near the membrane (5 μm) during a 3 ms heating pulse (Carvalho-de-Souza et al. 2015). To simulate the optocapacitive currents, *Vm* was set to − 60 mV and *Vs* to 130 mV in accordance with previously reported values (Shapiro et al. 2012). We can observe in Fig. 2 how the change in the temperature (Fig. 2D) generates the optocapacitive current (Fig. 2E). Since the heating and cooling of the membrane are governed by two different processes, respectively, the absorption of light and heat diffusion into the solution, $dT/dt$ is asymmetric, and so is the optocapacitive current. Due to the distance between the heating source and the membrane, the increase of temperature shows a lag (10–20 μs) and has a maximum $dT/dt$ at around 50 μs, which generates a rising phase in the optocapacitive current (insets in Fig. 2D and E). After the maximum, $dT/dt$ decreases with the consequent reduction of the optocapacitive current. Thus, the stereotypical shape of the optocapacitive currents with a rising phase, a peak, and a falling phase arises from the time course of $dT/dt$. The earliest account of such currents comes from experiments in which *Xenopus laevis* oocytes were exposed to temperature jumps induced by heating with a xenon lamp (Parker 1989). In response to these temperature jumps, a current associated with the displacement of charges in the membrane was observed, though the contribution of the membrane capacitance to this current was neglected due to their high reversal potential. Remarkably these currents are more pronounced when the black animal pole that contains melanin granules is illuminated.

The first detailed description of the optocapacitive currents and their mechanistic basis derives from the study of infrared light stimulation (Shapiro et al. 2012). Pulsed infrared light had been shown promising results as an optical noninvasive method for neuronal stimulation (Wells et al. 2005). Applying infrared light to *Xenopus* oocytes, mammalian cells, and lipid bilayers, Shapiro and cols. found that infrared light (~ 2 μm wavelength) generates a fast increase in temperature due to the absorption by water (Shapiro et al. 2012). This local increase in temperature leads to an increase in membrane capacitance in cells and even bilayers, demonstrating the general physical basis of this process. The voltage dependence of the optocapacitive currents is linear, and in mammalian cells and *Xenopus* oocytes, they have a highly positive reversal potential, but in painted lipid bilayers, the reversal potential is zero. This can be explained using Eq. 10, cells have a large surface potential difference between the internal and external faces, while painted bilayers have no such surface potential difference due to their symmetric composition. To further demonstrate the capacitive nature of the light-induced currents, polyvalent cations were used to manipulate the surface potential. Magnesium (Mg^+2^) or gadolinium (Gd^+3^) greatly altered the reversal potential of the optocapacitive current, consistent with the screening of the membrane surface charge by these ions (Shapiro et al. 2012).

The clear identification and isolation of optocapacitive currents in cells pose a challenge. In part because not only optocapacitive currents are elicited due to temperature changes, but currents can also arise due to other temperature-dependent phenomena. For example, an overall increase in the ionic conductance of channels present in the membrane or the activation of temperature dependent channels (Maingret 2000). However, we can use the unique properties of optocapacitive currents, namely their dependence on the rate of change of temperature, a highly positive reversal potential, and their asymmetric nature, to distinguish them from other temperature-sensitive currents.

## Electrical stimulation using optocapacitance

Light-induced neuronal remote modulation has revolutionized our understanding of electrical circuits in the brain and neurological diseases (Boyden et al. 2005; Li et al. 2005; Montagni et al. 2019; Tønnesen 2013). To achieve neuronal stimulation or inhibition, a mechanism that transduces light into an electrical signal is needed. In the case of optogenetics, channels that open with light have been used with great success (Fenno et al. 2011; Nagel et al. 2003; Valeeva et al. 2016). On the other hand, the depolarizing nature of the optocapacitive currents is especially well suited for the electrical stimulation of neurons due to their membrane composition and does not require genetic manipulation. In fact, in optocapacitance, the nature of depolarization is completely different from optogenetics. The initial membrane depolarization of optocapacitance is the result of a capacitive current related to the redistribution of charge near the membrane with no ionic conduction through the membrane involved, while the depolarization in optogenetics is produced by an ionic current, which may or may not have consequences in cell homeostasis.

We will first describe how optocapacitive currents can be used to generate a depolarization of the membrane in the absence of an external current source (current-clamp conditions with no imposed current). Based on the model shown in Fig. 1b, the current due to the ionic conductance (*I*_*i*_) in the membrane (through ion channels and transporters) relies on their conductance and reversal potential. This can be written in terms of the Thevenin equivalent by the following equation (Carvalho-de-Souza et al. 2017):

13 $${I}_{i}=\frac{\left(Vm-Vr\right)}{Rm}$$

Since there is no external current source acting on the cell membrane the net current should be equal to 0. Therefore,

14 $${I}_{C}+{I}_{i}=0$$

and

15 $$\left(Vm-Vs\right)\frac{dC}{dt}+C\frac{{dV}_{m}}{dt}+\frac{\left(Vm-Vr\right)}{Rm}=0$$

16 $$\frac{{dV}_{m}}{dt}=-\left(\frac{\left(Vm-Vr\right)}{Rm C\left(t\right)}+\frac{\left(Vm-Vs\right)}{C\left(t\right)}\frac{dC}{dt}\right)$$

From Eq. 16, we can note that, assuming a constant *Rm* and knowing the capacitance time course, we can solve the differential equation for *Vm*. As we showed previously, the capacitance can be changed by temperature, and due to the large and positive values of *Vs*, changes in capacitance will generate a depolarization. An important result from this equation shows that a faster change in capacitance (*dC*/*dt*) generates a larger change in the voltage (*dVm*/*dt*), thus, being more effective in triggering action potentials. The assumption of a constant *Rm* works well for stimulation times in the microsecond scale, which is faster than ion channel kinetics. Such short times can be easily achieved (Carvalho-de-Souza et al. 2017). If the temperature is changed slowly, it will most likely not elicit an action potential but rather disturb the behavior of ion channels and transporters (Shapiro et al. 2013), possibly impairing the normal functioning of excitable cells (Ait Ouares et al. 2019; Owen et al. 2019). Considering Eq. 16, if the total capacitance C increases, meaning a larger cell, to achieve the same depolarization, *dC*/*dt* must be larger. This could be achieved either by heating a larger area of the membrane or making a faster *dT*/*dt*. Membrane lipidic composition varies greatly among cells (Symons et al. 2021). These different compositions could affect the surface charge of the inner or outer leaflet, which would be reflected in different *Vs*. Even tough membrane composition can change, the surface charge asymmetry seems to be conserved in eukaryotic cells (Doktorova et al. 2020), thus *Vs* is expected to be positive for most cell types. Even if *Vs* changes from different cell types, the change would be in the amplitude of the depolarized current, rather than the rate of the change of capacitance (*dC*/*dt*).

For optocapacitive stimulation of neurons ideally, the heating should be fast and restricted to the membrane. An exceptionally useful tool to achieve this is the use of light-absorbing nanomaterials as photothermal transducers that convert electromagnetic radiation to heat at the membrane. Among such nanomaterials, we find gold nanoparticles and nanorods, carbon nanotubes, graphite nanoparticles (Carvalho-de-Souza et al. 2017, 2015), silicon-based nanomaterials (Fang et al. 2018; Jiang et al. 2018, 2016), graphene derivatives (Rastogi et al. 2020), and titanium carbide metal flakes (Wang et al. 2021). This variety of photothermal transducers provides an array of different chemical, mechanical and optical properties for remote neuronal stimulation using optocapacitance. Moreover, the ability to conjugate nanoparticles to antibodies, toxins (Carvalho-de-Souza et al. 2015), or small molecules such as cholesterol (Carvalho-de-Souza et al. 2019) can provide stimulation directed to a subpopulation of cells and restrict the heating to the membrane. The variety of physical properties of nanoparticles and their modifications will affect the adsorption of nanoparticles in the cell membrane, thus modulating the efficiency of the optocapacitive effect (Mu et al. 2014). Due to heat diffusion, nanoparticles in close contact with the membrane are more efficient at generating optocapacitive stimulation than loosely bound ones (Carvalho-de-Souza et al. 2015). At present, nanoparticles have been used to elicit optocapacitive stimulation of dorsal root ganglion neurons (DRG) in culture, hippocampal slices, and organoids (Carvalho-de-Souza et al. 2015; Rastogi et al. 2020) and in the isolated retina (Carvalho de Souza et al. personal communication). These promising results are presently being extended to in vivo models.

**Fig. 3:**
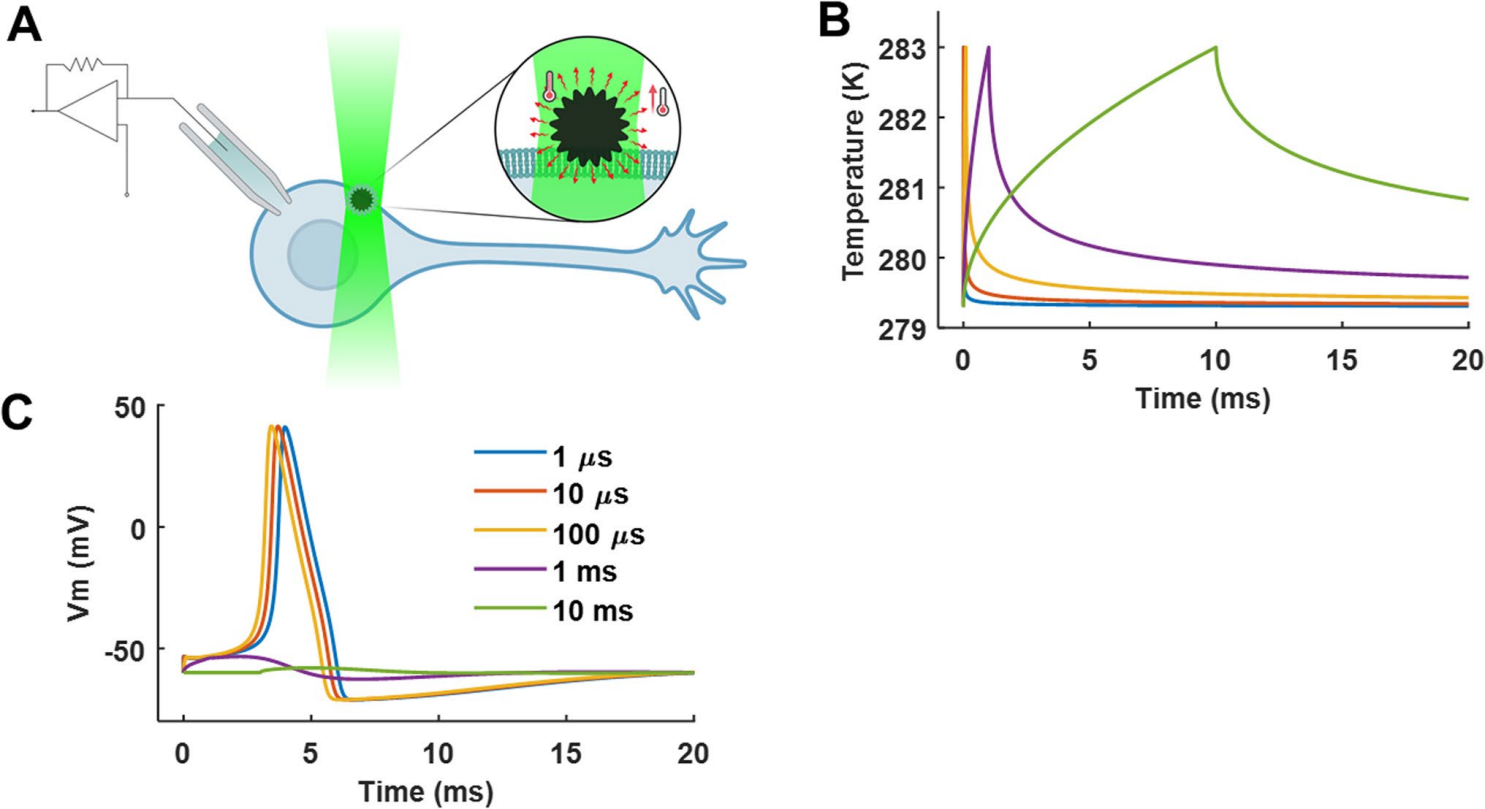
Optocapacitive generation of action potentials. **A** diagram of a neuron in whole-cell patch-clamp configuration interfaced with a photothermal transducer under green laser illumination. **B** and **C** are, respectively, the simulated temperature change (3.7 °C) and membrane voltage response for different times of stimulation (a legend in **C**)

To exemplify the possibilities that optocapacitance offers in firing action potentials, we provide a simulation of optocapacitive stimulation through the illumination of a photothermal transducer in close proximity to a neuron, as previously described (Carvalho-de-Souza et al. 2015) (Fig. 3a). The neuron is simulated using the biophysical properties of the squid axon membrane described by the Hodgkin and Huxley model with the addition of optocapacitive currents (Hodgkin and Huxley 1952). The heating source produces a change in temperature proportional to $\sqrt t$, which provides a good approximation for nanoparticle-mediated heating (Carvalho-de-Souza et al. 2017). A change in temperature of 3.7 °C was applied over different time spans (1 μs to 10 ms) (Fig. 3b). This simple model reproduces the effect observed in DRG neurons (Carvalho-de-Souza et al. 2015). Also, it can be observed that only when the temperature change occurs over a short period of time (1 to 100 μs) the cell fires an action potential. For longer times, only subthreshold depolarization is observed. Since the final temperature reaches the same value for the different stimulation times, this shows that it is not the final temperature or the final capacitance that matters to reach the threshold voltage and generate an action potential. Rather, it is the rate of change in temperature and consequently the rate of change in capacitance that is relevant.

## Addressing the optocapacitive mechanism of electrical stimulation

When stimulating neurons with light, it is necessary to distinguish optocapacitive effects from other temperature-dependent phenomena. Since optocapacitive currents depend on the physical properties of the membrane, they cannot be eliminated or affected without compromising the integrity of the membrane or its basic physical properties. To address the role of optocapacitance in the stimulation of neurons, we can rely on the relationship between energy (*E*_Th_) needed to reach the threshold voltage (*V*_Th_) for an action potential and the time of stimulation (Δ*t*) (Carvalho-de-Souza et al. 2017). The relation between *E*_Th_ and Δ*t* for the optocapacitive effect is given by the following equation (Carvalho-de-Souza et al. 2017):

17 $$E_{Th}=\frac{V_R-V_{Th}}{\gamma c(V_{Th}-V_s)}\sqrt{\triangle t}$$

where *c* is a proportionality constant and γ is the factor that defines the linear relationship between capacitance and temperature (~ 0.01 °C^−1^). This equation indicates that the energy necessary to stimulate a neuron increases with the square root of the stimulation time. Thus, applying more energy in less time provides a more efficient way to stimulate neurons. This means that for any given stimulation, applying half of the energy in a fourth of the time will generate the same depolarization. This has an appealing advantage since, in the time frame of microseconds, the heat generated by the nanoparticles will not affect ion channels and transporters that are usually activated in the millisecond range (Hodgkin and Huxley 1952). Therefore, the optocapacitive currents would dominate at short stimulation times. The relationship between energy and stimulation time is a benchmark of optocapacitive neuronal stimulation. This has been tested in DRG neurons where *E*_Th_ and Δ*t* have a power relationship with an exponent of ~ 0.6–0.8, instead of the 0.5 predicted by Eq. 17 (Carvalho-de-Souza et al. 2019, 2017; Rastogi et al. 2020). This discrepancy can be explained by the assumptions and simplifications of this equation, namely the consideration of infinite membrane resistance and a homogeneous distribution of nanoparticles in respect to the membrane. Considering this, a power relation between *E*_Th_ and Δ*t* provides strong evidence of an underlying optocapacitance mediated stimulation.

## Challenges for in vivo optocapacitive electrical stimulation

Even though much progress has been made in the field of neuronal stimulation by optocapacitance, there are still several requirements for its applicability in vivo. These involve mainly (1) the delivery of photothermal transducers, (2) achievement of fast and localized illumination, and (3) tissue penetration.

The application of nanoparticles in vivo faces similar challenges as those encountered for the use of optogenetic opsins, which requires the local delivery of adeno-associated viral vectors. These challenges include immune responses, specialized methods for delivery or transport to the target cells, and clearance (Shen et al. 2020). The biocompatibility of photothermal transducers has been tackled mostly in vitro, but it remains to be thoroughly assessed in vivo (Jiang et al. 2016; Rastogi et al. 2020; Wang et al. 2021). Studies have shown that after systemic delivery through injection into the bloodstream, gold nanoparticles have poor penetration into the brain, especially for nanoparticles over 15 nm (De Jong et al. 2008; Sonavane et al. 2008). Non-invasive delivery systems for the central nervous system will require photothermal transducers to be able to pass through the blood–brain barrier (BBB). This will most likely involve the conjugation of nanoparticles to molecules that can be recognized and promote their uptake by the BBB (Pinheiro et al. 2021). Alternatively, the delivery of nanoparticles could be achieved by the retrograde transport from nerve terminals. The use of fluorescent latex nanoparticles for the study of retrograde transport in vitro and in vivo shows that nanoparticles can be uptaken by terminals and be transported into the cell body (Katz et al. 1984). Since the optocapacitive effect can be triggered from nanoparticles on the inside of the cell (Carvalho-de-Souza et al. 2019), retrograde transport provides a promising method for delivery and stimulation in vivo. To prevent unwanted effects on the target tissue, a homogeneous dispersion of nanoparticles is necessary; aggregation of nanoparticles can generate hotspots that can damage the cell membrane. Aggregation of nanoparticles can be prevented by surface modifications that enhance stability in solution, such as PEGylation (Zhang et al. 2009). In order to achieve a controlled dosage of nanoparticles and thus reliable photostimulation, an effective delivery process must be achieved.

For an efficient generation of optocapacitive currents, delivery of high-intensity light pulses is necessary. This can be achieved using pulsed lasers, laser diodes, or high-power LEDs, these light sources are not easily implantable in vivo, but they can be delivered via fiber optics. The development of implantable micrometer size LED devices could pave the way for brain stimulation (Gutruf et al. 2019; Wu et al. 2015).

Lastly, stimulation of deep parts of the brain remains challenging due to the low penetration of light in tissues (Hong et al. 2017). Therefore, it is necessary to use other wavelengths of the electromagnetic radiation spectrum, such as near infrared, which may penetrate as deep as 1 cm. Alternatively, radiofrequency in the low GHz range or alternating magnetic fields may be used due to their better penetration in tissues (Gabriel et al. 1996a, b; Lim et al. 2003; Young et al. 1980). However, to achieve the same stimulation efficiency of light and nanoparticles, deep brain stimulation using optocapacitance will require the development of appropriate thermal transducers materials for the sought radio frequencies. Magnetic-field heating nanoparticles could be well suited for deep brain stimulation even though currently the heating is too slow to obtain an optocapacitive effect (Chen et al. 2015; Huang et al. 2010; Roet et al. 2019). We believe that addressing these issues systematically will provide new avenues for remote stimulation without the need for genetic manipulation in vivo.

## References

[CR1] Ait Ouares K, Beurrier C, Canepari M, Laverne G, Kuczewski N (2019). Opto nongenetics inhibition of neuronal firing. Eur J Neurosci.

[CR2] Bondelli G, Sardar S, Chiaravalli G, Vurro V, Paternò GM, Lanzani G, D’Andrea C (2021). Shedding light on thermally induced optocapacitance at the organic biointerface. J Phys Chem B.

[CR3] Boyden ES, Zhang F, Bamberg E, Nagel G, Deisseroth K (2005). Millisecond-timescale, genetically targeted optical control of neural activity. Nat Neurosci.

[CR4] Carvalho-de-Souza JL, Nag OK, Oh E, Huston AL, Vurgaftman I, Pepperberg DR, Bezanilla F, Delehanty JB (2019). Cholesterol functionalization of gold nanoparticles enhances photoactivation of neural activity. ACS Chem Neurosci.

[CR5] Carvalho-de-Souza JL, Pinto BI, Pepperberg DR, Bezanilla F (2017). Optocapacitive generation of action potentials by microsecond laser pulses of nanojoule energy. Biophys J.

[CR6] Carvalho-de-Souza JL, Treger JS, Dang B, Kent SBH, Pepperberg DR, Bezanilla F (2015). Photosensitivity of neurons enabled by cell-targeted gold nanoparticles. Neuron.

[CR7] Chen R, Romero G, Christiansen MG, Mohr A, Anikeeva P (2015). Wireless magnetothermal deep brain stimulation. Science.

[CR8] Daleke DL, Huestis WH (1985). Incorporation and translocation of aminophospholipids in human erythrocytes. Biochemistry.

[CR9] De Jong WH, Hagens WI, Krystek P, Burger MC, Sips AJAM, Geertsma RE (2008). Particle size-dependent organ distribution of gold nanoparticles after intravenous administration. Biomaterials.

[CR10] Doktorova M, Symons JL, Levental I (2020). Structural and functional consequences of reversible lipid asymmetry in living membranes. Nat Chem Biol.

[CR11] Fang Y, Jiang Y, Acaron Ledesma H, Yi J, Gao X, Weiss DE, Shi F, Tian B (2018). Texturing silicon nanowires for highly localized optical modulation of cellular dynamics. Nano Lett.

[CR12] Fenno L, Yizhar O, Deisseroth K (2011). The development and application of optogenetics. Annu Rev Neurosci.

[CR13] Gabriel C, Gabriel S, Corthout E (1996). The dielectric properties of biological tissues: I. Literature survey. Phys Med Biol.

[CR14] Gabriel S, Lau RW, Gabriel C (1996). The dielectric properties of biological tissues: II. Measurements in the frequency range 10 Hz to 20 GHz. Phys Med Biol.

[CR15] Gutruf P, Yin RT, Lee KB, Ausra J, Brennan JA, Qiao Y, Xie Z, Peralta R, Talarico O, Murillo A, Chen SW, Leshock JP, Haney CR, Waters EA, Zhang C, Luan H, Huang Y, Trachiotis G, Efimov IR, Rogers JA. 2019. Wireless, battery-free, fully implantable multimodal and multisite pacemakers for applications in small animal models. Nat Commun 10.10.1038/s41467-019-13637-w 10.1038/s41467-019-13637-w PMC6917818 31848334

[CR16] Hodgkin AL, Huxley AF (1952). A quantitative description of membrane current and its application to conduction and excitation in nerve. J Physiol.

[CR17] Hong G, Antaris AL, Dai H. 2017. Near-infrared fluorophores for biomedical imaging.Nat Biomed Eng 1.10.1038/s41551-016-0010

[CR18] Huang H, Delikanli S, Zeng H, Ferkey DM, Pralle A (2010). Remote control of ion channels and neurons through magnetic-field heating of nanoparticles. Nat Nanotechnol.

[CR19] Jiang Y, Carvalho-De-Souza JL, Wong RCS, Luo Z, Isheim D, Zuo X, Nicholls AW, Jung IW, Yue J, Liu DJ, Wang Y, De Andrade V, Xiao X, Navrazhnykh L, Weiss DE, Wu X, Seidman DN, Bezanilla F, Tian B (2016). Heterogeneous silicon mesostructures for lipid-supported bioelectric interfaces. Nat Mater.

[CR20] Jiang Y, Li X, Liu B, Yi J, Fang Y, Shi F, Gao X, Sudzilovsky E, Parameswaran R, Koehler K, Nair V, Yue J, Guo KH, Fang Y, Tsai HM, Freyermuth G, Wong RCS, Kao CM, Chen CT, Nicholls AW, Wu X, Shepherd GMG, Tian B (2018). Rational design of silicon structures for optically controlled multiscale biointerfaces. Nat Biomed Eng.

[CR21] Katz LC, Burkhalter A, Dreyer WJ (1984). Fluorescent latex microspheres as a retrograde neuronal marker for in vivo and in vitro studies of visual cortex. Nature.

[CR22] Kučerka N, Nieh MP, Katsaras J (2011). Fluid phase lipid areas and bilayer thicknesses of commonly used phosphatidylcholines as a function of temperature. Biochim Biophys Acta Biomembr.

[CR23] Li X, Gutierrez DV, Hanson MG, Han J, Mark MD, Chiel H, Hegemann P, Landmesser LT, Herlitze S (2005). Fast noninvasive activation and inhibition of neural and network activity by vertebrate rhodopsin and green algae channelrhodopsin. Proc Natl Acad Sci U S A.

[CR24] Lim YT, Kim S, Nakayama A, Stott NE, Bawendi MG, Frangioni JV (2003). Selection of quantum dot wavelengths for biomedical assays and imaging. Mol Imaging.

[CR25] Maingret F (2000). TREK-1 is a heat-activated background K+ channel. EMBO J.

[CR26] Martino N, Feyen P, Porro M, Bossio C, Zucchetti E, Ghezzi D, Benfenati F, Lanzani G, Antognazza MR (2015). Photothermal cellular stimulation in functional bio-polymer interfaces. Sci Rep.

[CR27] McLaughlin S (1989). The electrostatic properties of membranes. Annu Rev Biophys Biophys Chem.

[CR28] Montagni E, Resta F, Mascaro ALA, Pavone FS (2019). Optogenetics in brain research: from a strategy to investigate physiological function to a therapeutic tool. Photonics.

[CR29] Mu Q, Jiang G, Chen L, Zhou H, Fourches D, Tropsha A, Yan B (2014). Chemical basis of interactions between engineered nanoparticles and biological systems. Chem Rev.

[CR30] Nagel G, Szellas T, Huhn W, Kateriya S, Adeishvili N, Berthold P, Ollig D, Hegemann P, Bamberg E (2003). Channelrhodopsin-2, a directly light-gated cation-selective membrane channel. Proc Natl Acad Sci.

[CR31] Owen SF, Liu MH, Kreitzer AC (2019). Thermal constraints on in vivo optogenetic manipulations. Nat Neurosci.

[CR32] Parker I (1989). Ionic and charge-displacement currents evoked by temperature jumps in xenopus oocytes. Proc R Soc London B Biol Sci.

[CR33] Petrache HI, Dodd SW, Brown MF (2000). Area per lipid and acyl length distributions in fluid phosphatidylcholines determined by 2H NMR spectroscopy. Biophys J.

[CR34] Pinheiro RGR, Coutinho AJ, Pinheiro M, Neves AR (2021). Nanoparticles for targeted brain drug delivery: what do we know?. Int J Mol Sci.

[CR35] Pinto B, Bassetto CAZ, Latorre R, Bezanilla F (2021). Measuring temperature time course using membrane capacitance. Biophys J.

[CR36] Plaksin M, Shapira E, Kimmel E, Shoham S (2018) Thermal transients excite neurons through universal intramembrane mechanoelectrical effects.Phys Rev X 8.10.1103/PhysRevX.8.011043

[CR37] Rastogi SK, Garg R, Scopelliti MG, Pinto BI, Hartung JE, Kim S, Murphey CGE, Johnson N, Roman DS, Bezanilla F, Cahoon JF, Gold MS, Chamanzar M, Cohen-Karni T (2020). Remote nongenetic optical modulation of neuronal activity using fuzzy graphene. Proc Natl Acad Sci U S A.

[CR38] Roet M, Hescham SA, Jahanshahi A, Rutten BPF, Anikeeva PO, Temel Y (2019). Progress in neuromodulation of the brain: a role for magnetic nanoparticles?. Prog Neurobiol.

[CR39] Seigneuret M, Devaux PF (1984). ATP-dependent asymmetric distribution of spin-labeled phospholipids in the erythrocyte membrane: relation to shape changes. Proc Natl Acad Sci U S A.

[CR40] Shapiro MG, Homma K, Villarreal S, Richter C-P, Bezanilla F (2012). Infrared light excites cells by changing their electrical capacitance. Nat Commun.

[CR41] Shapiro MG, Priest MF, Siegel PH, Bezanilla F (2013). Thermal mechanisms of millimeter wave stimulation of excitable cells. Biophys J.

[CR42] Shen Y, Campbell RE, Côté DC, Paquet ME (2020). Challenges for therapeutic applications of opsin-based optogenetic tools in humans. Front Neural Circuits.

[CR43] Sonavane G, Tomoda K, Makino K (2008). Biodistribution of colloidal gold nanoparticles after intravenous administration: effect of particle size. Colloids Surf B Biointerfaces.

[CR44] Symons JL, Cho KJ, Chang JT, Du G, Waxham MN, Hancock JF, Levental I, Levental KR (2021). Lipidomic atlas of mammalian cell membranes reveals hierarchical variation induced by culture conditions, subcellular membranes, and cell lineages. Soft Matter.

[CR45] Szekely P, Dvir T, Asor R, Resh R, Steiner A, Szekely O, Ginsburg A, Mosenkis J, Guralnick V, Dan Y, Wolf T, Tamburu C, Raviv U (2011). Effect of temperature on the structure of charged membranes. J Phys Chem B.

[CR46] Taylor RE (1965). Impedance of the squid axon membrane. J Cell Comp Physiol.

[CR47] Tønnesen J (2013). Optogenetic cell control in experimental models of neurological disorders. Behav Brain Res.

[CR48] Valeeva G, Tressard T, Mukhtarov M, Baude A, Khazipov R (2016). An optogenetic approach for investigation of excitatory and inhibitory network GABA actions in mice expressing channelrhodopsin-2 in GABAergic neurons. J Neurosci.

[CR49] Wang Y, Garg R, Hartung JE, Goad A, Patel DA, Vitale F, Gold MS, Gogotsi Y, Cohen-Karni T (2021). Ti3C2TxMXene flakes for optical control of neuronal electrical activity. ACS Nano.

[CR50] Wells J, Kao C, Mariappan K, Albea J, Jansen ED, Konrad P, Mahadevan-Jansen A (2005). Optical stimulation of neural tissue in vivo. Opt Lett.

[CR51] Wu F, Stark E, Ku PC, Wise KD, Buzsáki G, Yoon E (2015). Monolithically Integrated μLEDs on silicon neural probes for high-resolution optogenetic studies in behaving animals. Neuron.

[CR52] Young JH, Wang MT, Brezovich IA (1980). Frequency/depth-penetration considerations in hyperthermia by magnetically induced currents. Electron Lett.

[CR53] Zhang G, Yang Z, Lu W, Zhang R, Huang Q, Tian M, Li L, Liang D, Li C (2009). Influence of anchoring ligands and particle size on the colloidal stability and in vivo biodistribution of polyethylene glycol-coated gold nanoparticles in tumor-xenografted mice. Biomaterials.

